# Relationship between single-nucleotide polymorphisms and cancer immunotherapy efficacy and toxicity: a systematic review

**DOI:** 10.3389/fonc.2025.1653990

**Published:** 2025-10-31

**Authors:** Monia Specchia, Marco Siringo, Eva Mazzotti, Federica Mazzuca

**Affiliations:** ^1^ Oncology Unit, Sant’Andrea University Hospital, Rome, Italy; ^2^ Department of Clinical and Molecular Medicine, Sapienza University of Rome, Rome, Italy; ^3^ Department of Molecular Medicine, Sapienza University of Rome, Rome, Italy

**Keywords:** SNP, SNPS, single nucleotide polymorphisms, immunotherapy, cancer

## Abstract

Immunotherapy has revolutionized cancer treatment by using the body’s immune system to target and eliminate tumor cells. Immune checkpoint inhibitors (ICIs), such as anti-PD-1/PD-L1 and anti-CTLA-4 therapies, have shown substantial clinical benefits in many types of cancer. Despite their efficacy, not all patients benefit from them, and there is a need to identify biomarkers to predict responses and adverse events. This systematic review explores the role of single nucleotide polymorphisms (SNPs) in cancer immunotherapy, focusing on genes involved in immune checkpoint regulation. A comprehensive literature search was conducted across two databases, PubMed and Cochrane, published from 2000 to 2024, for a total of 884 works. The final analysis included 29 records that assessed the impact of SNPs on immunotherapy responses and toxicities. Findings suggest that specific SNPs in the CTLA-4, PD-1, and PD-L1 genes influence both treatment outcomes and the risk of immune-related adverse events across various cancers. For instance, certain CTLA-4 and PD-1 SNPs were associated with better survival rates or higher toxicity risks, while PD-L1 SNPs influenced tumor responses to ICIs. Specific SNPs, such as those in the CTLA-4 and PD-1 genes, have been linked to improved survival or increased toxicity risk. Additionally, PD-L1 SNPs can impact tumor response to ICIs, offering insights into their potential as predictive biomarkers. The findings emphasize the importance of SNPs in personalized cancer therapy, enabling more effective and safer treatment strategies. However, further research is needed to validate these genetic markers and optimize their clinical utility in immunotherapy.

## Introduction

Immunotherapy has totally revolutionized cancer treatment by harnessing the body’s immune system to target and eliminate malignant cells. Unlike traditional therapies such as chemotherapy and radiotherapy, immunotherapy is able to activate either passive or active immunity to target and destroy tumor cells. A critical factor influencing tumor progression is the tumor microenvironment (TME), which contributes to immune evasion mechanisms that enable tumors to escape immune surveillance. Several cancer immunotherapies, including immune checkpoint inhibitors (ICIs), cancer vaccines, and adoptive cell transfer (ACT), have shown remarkable efficacy, including positive response rates, prolonged time to response, and, in most cases, good tolerability (1). However, not all patients respond to immunotherapy, and some experience varying adverse effects, which are not always predictable and can be challenging to manage.

ICIs, particularly those targeting PD-1/PD-L1 (programmed Death protein 1/programmed Death-Ligand 1) pathway and CTLA-4 (cytotoxic T-lymphocyte associated protein 4), have changed cancer treatment paradigm, offering significant clinical benefits in various cancer types, including melanoma, non-small cell lung cancer (NSCLC), kidney cancer and many others (2). Despite their success, the response to ICIs is heterogeneous, and no current biomarkers are still available. For example, tissue PD-L1 expression detected by immunohistochemistry (IHC) in some cancer histology, is not consistently predictive due to variability in assay methods and interpretation (3).

As a consequence, there is an urgent need to identify biomarkers that can predict the likelihood of a patient benefiting from immunotherapy and the potential for developing serious adverse effects. One promising avenue for such biomarker discovery involves the study of single nucleotide polymorphisms (SNPs) in genes involved in immune response mechanisms, because they influence immune system function and may contribute to both the efficacy of immunotherapies and the occurrence of adverse reactions (4). In fact, SNPs that affect immune system genes, particularly those involved in immune checkpoint regulation, may provide a more reliable and personalized approach to predicting treatment outcomes (5–7).

This systematic review aims to explore the role of SNPs in immunotherapy, focusing on genetic variants influence in immune responses, treatment efficacy, and development of adverse effects. By understanding the genetic underpinnings of immunotherapy responses, we can move toward more tailored, effective, and safer treatment strategies for cancer patients.

## Materials and methods

We conducted a systematic analysis of SNPs, focusing on individual genes categorized by their receptor mechanisms, rather than grouping them by specific diseases.

Our research methodology included studies, by gene type, selecting relationships with prognostic factors and adverse effects. However, all SNPs references can be searched on the dbSNP platform, National Library of Medicine, which we did not use for our purpose.

The search was conducted in adherence to the Preferred Reporting Items for Systematic Reviews and Meta-Analyses (PRISMA) guidelines (8), focusing on studies published between 2000 and November 2024.

### Eligibility criteria

We included studies published in English, including both animal and human preclinical studies, as well as retrospective and prospective clinical studies, that addressed the role of SNPs in the context of ICIs.

### Information sources

A comprehensive literature search was performed on PubMed and Cochrane to identify published articles that explore the impact of SNPs on the response and toxicities associated with ICIs in cancer treatment. If multiple studies reported the same findings, only the most recent publication was retained to avoid duplication. Conference abstracts were also included if they provided relevant and original data within the scope of this review. Exclusion criteria comprised duplicate reports of the same patient cohorts, and editorials or commentaries deemed irrelevant to the objectives of this review.

### Search strategy

The search strategy included the following terms: (“SNP” OR “SNPs” OR “single nucleotide polymorphisms”) AND (“immunotherapy” AND “cancer”).

**Figure 1:**
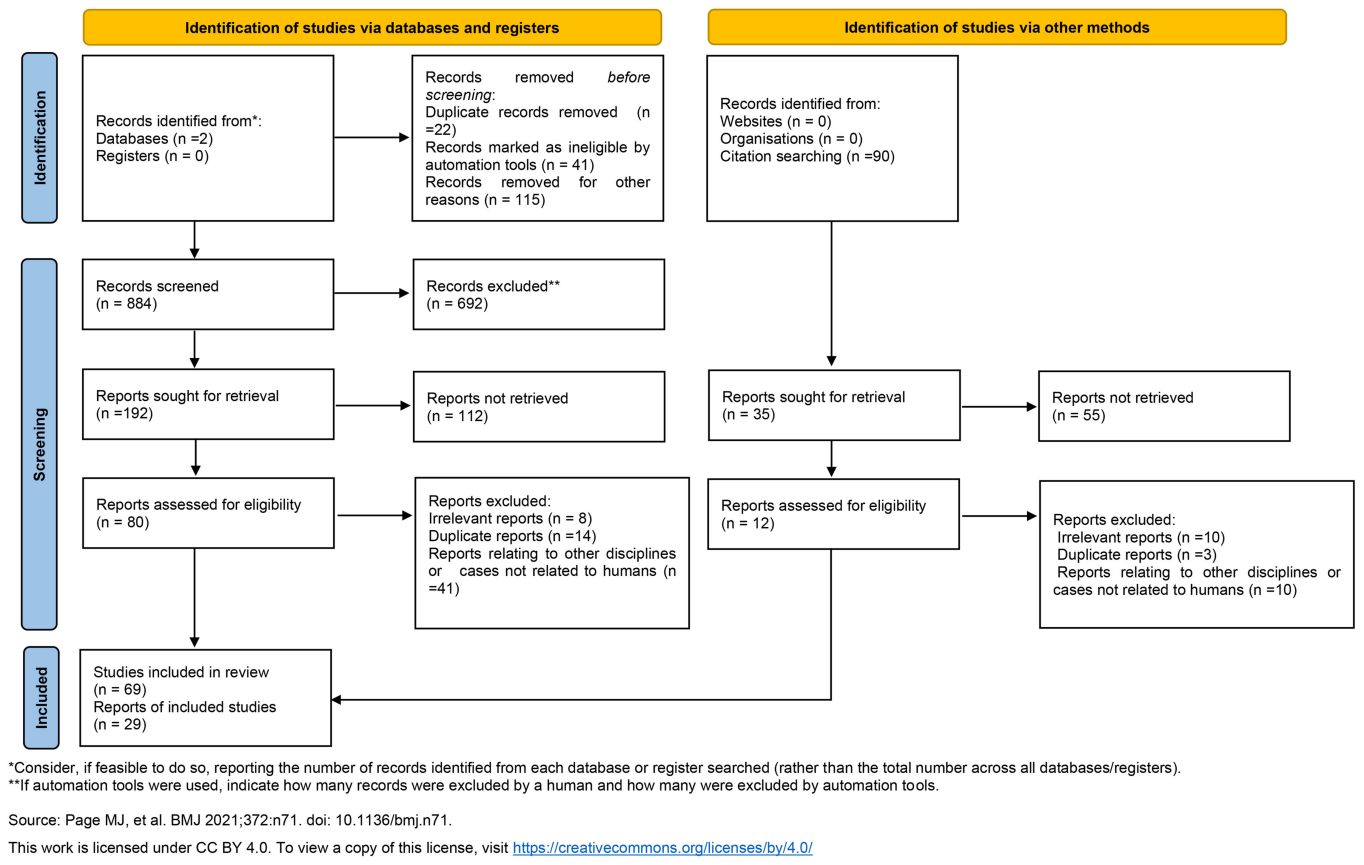
The review flow diagram (PRISMA 2020).

All identified records were independently screened by two authors (MS and MS), who reviewed the abstracts for relevance. Following this, full-text articles were examined for eligibility. A total of 29 records were identified through the literature search. In addition, relevant articles that were not initially captured by the search strategy were included in the analysis. The PRISMA 2020 flow diagram outlining the search strategy and selection process is presented in **Figure 1**.

## Results

### CTLA-4 gene polymorphisms

CTLA-4 is a crucial immune checkpoint receptor that belongs to the immunoglobulin receptor superfamily. It plays a key role in regulating T-cell activation by inhibiting costimulatory signals from CD28, ultimately dampening the immune response. This mechanism is also exploited by tumors to evade immune detection, as cancer cells can upregulate CTLA-4, leading to suppressed immune responses and promoting tumor growth (9).

Several SNPs in the CTLA-4 gene have been investigated as predictive biomarkers of immunotherapy outcomes. Importantly, these SNPs may modify CTLA-4 expression or activity by influencing transcription factor binding, mRNA splicing, or protein function.

In a large multicentre study involving 361 melanoma patients treated with ipilimumab across six hospitals in Switzerland and the Netherlands, the relationship between 10 CTLA-4 SNPs and treatment outcomes was explored. The results revealed that specific CTLA-4 SNPs could help predict both adverse events (AEs) and overall survival (OS). For example, the TT genotype of the -1722T>C SNP was associated with a lower incidence of grade ≥3 AEs (p=0.049), while the GG genotype of the CT60G>A SNP correlated with a higher risk of severe AEs (p=0.026). Additionally, the TT genotype of the Jo27T>C SNP (p=0.056) and the GG genotype of the Jo31G>T SNP (p= 0.046) were associated to longer OS (10).

CTLA-4 SNPs can alter gene expression through multiple mechanisms: variants located in promoter regions (e.g., -1722T>C, c.-1661A>G) may modify transcription factor binding sites, and changing transcriptional activity; others, such as CT60G>A (rs3087243), influence alternative splicing and the ratio of soluble versus membrane-bound isoforms of CTLA-4, impacting T-cell inhibition.

In a separate large case-control study conducted in China, SNPs in the CTLA-4 immune checkpoint pathway were examined in relation to colorectal cancer risk and survival. This study, which included over 1,000 patients, found that individuals carrying the A allele of B7–2 rs2681416 had a significantly increased risk of colorectal cancer, especially colon cancer. The rs2681416 variant was also associated with poorer survival in colon cancer patients, and it influenced the expression of the IQCB1 gene, which modulates immune cell infiltration (Th17 cells) in the tumor microenvironment. This research highlights how CTLA-4 SNPs may impact both cancer susceptibility and immune system activity (11).

Furthermore, the CTLA-4c.-1661A>G SNP has been shown to create a binding site for the C/EBPβ transcription factor, leading to increased CTLA-4 expression. This variant could be a potential risk factor for certain cancers, particularly gastric and breast cancer. Similarly, the rs3087243G>A (CTLA-4CT60G>A) SNP has been associated with an increased risk of skin cancer, while other studies have linked this SNP to a higher risk of cervical and breast cancers (12, 13).

Additionally, an analysis of seven SNPs (rs733618, rs4553808, rs11571317, rs5742909, rs231775, rs3087243, and rs7565213) in melanoma patients treated with CTLA-4 blockade revealed that specific SNPs, such as rs4553808, rs11571327, and rs231775, were linked to treatment response. The TGCCAGG haplotype was associated with a positive response to therapy, while the TACCGGG haplotype was associated with no response. However, no significant relationship was found between these SNPs and the occurrence of severe autoimmune reactions (14).

In conclusion, CTLA-4 gene polymorphisms have emerged as potential biomarkers for predicting both cancer risk and treatment outcomes in immunotherapy. These SNPs may influence immune responses and help determine a patient’s likelihood of responding to treatment or developing adverse effects.

### PD-1 gene polymorphisms

PD-1 is an immune checkpoint receptor that is a type I transmembrane protein within the immunoglobulin superfamily. It is expressed in various immune cell types, including CD4+ and CD8+ T cells, B cells, macrophages, natural killer T (NKT) cells, and certain subsets of dendritic cells. Within the TME, the interaction between PD-1 and its ligand PD-L1, expressed on tumor cells, is a key mechanism of immune evasion, enabling tumor cells to escape immune surveillance (15).

PD-1 is encoded by the PDCD1 gene, located on chromosome 2q37.3, and plays a central role in regulating T cell responses and maintaining immune tolerance (16).

While anti-PD-1 therapies, such as nivolumab and pembrolizumab, have demonstrated significant efficacy in cancer treatment, not all patients respond to these therapies, and some experience severe immune-related adverse events (irAEs). Consequently, identifying predictive biomarkers to forecast treatment outcomes and toxicity is essential for optimizing therapy. Recent studies have shown positive results on the potential role of SNPs in the PDCD1 gene as predictive biomarkers of response to anti-PD-1 therapies.

In a 2021 Australian study, plasma DNA from patients with advanced melanoma who were treated with anti-PD-1 antibodies (nivolumab or pembrolizumab) was analysed for five specific PD-1 SNPs: PD1.1 (rs36084323, G>A), PD1.3 (rs11568821, G>A), PD1.5 (rs2227981, C>T), PD1.6 (rs10204225, G>A), and PD1.9 (rs2227982, C>T). This study found that patients with the G/G genotype of PD1.3 (rs11568821) had a higher rate of complete responses (16.5% vs. 2.6%) compared to those with the A/G genotype. Additionally, the G allele of PD1.3 was significantly associated with longer PFS (14.1 months vs. 7.0 months for the AG genotype) (p=0.04). No significant associations were found for the other SNPs with response, PFS, or OS (17).Another study examining the PDCD1 804C>T (rs2227981) SNP in patients with metastatic melanoma treated with anti-PD-1 monotherapy found that carriers of the T allele had significantly shorter OS compared to wild-type patients. The 3-year OS rate was 51.8% for T allele carriers, compared to 71% in wild-type patients (hazard ratio [HR] = 2.37; 95% CI: 1.11-5.04; *p* = 0.026). Furthermore, T allele carriers had a reduced fraction of peripheral PD-1+CD4+ T cells, which may influence the clinical benefit of PD-1 inhibition (18).

An Italian study evaluated the effects of five PD-1 SNPs (PD1.3 G>A, PD1.5 C>T, PD1.6 G>A, PD1.7 T>C, and PD1.10 C>G) and three PD-L1 SNPs (+8293 C>A, PD-L1 C>T, and PD-L1 G>C) in metastatic melanoma patients treated with nivolumab or pembrolizumab. The study observed that patients with the PD-L1 + 8293 C/A genotype had a reduced risk of irAEs compared to those with the C/C genotype (risk ratio = 0.45; 95% CI 0.22-0.93; *p* = 0.031). Additionally, a trend towards reduced irAEs was noted in patients carrying the PD1.5 T allele, and the PD1.7 C/C genotype was associated with a survival benefit (HR = 0.37; 95% CI 0.14-0.96; *P* = 0.028) (19).

In a separate study involving renal cancer patients treated with nivolumab, the effect of three PDCD1 SNPs (PD1.3, PD1.5, and PD1.6) on irAEs was assessed. The results indicated that patients with the G allele of PD1.6 (rs10204225) experienced more severe irAEs than those with the AA genotype (odds ratio = 3.39; 95% CI 1.52-7.76; *p* = 0.003), suggesting a potential association between PD-1 polymorphisms and the development of toxicity in patients treated with anti-PD-1 therapies for renal cancer (20).

From a functional perspective, PD-1 SNPs such as PD1.3 (rs11568821) are located in enhancer regions and may disrupt binding of transcription factors like RUNX1, leading to reduced PD-1 expression. Similarly, PD1.5 (rs2227981) and PD1.6 (rs10204225) have been associated with changes of PD-1+ T-cell subsets.

### PD-L1 gene polymorphisms

PD-L1 is frequently expressed in various human cancers, where it interacts with the PD-1 receptor on activated T cells, inhibiting antitumor immunity. This interaction effectively counteracts T-cell activation signals, contributing to immune evasion by tumor cells. The development of antibody-based inhibitors targeting the PD-1/PD-L1 pathway has led to significant clinical success in treating various cancers, making PD-L1 expression on tumor cells and other cells in the tumor microenvironment highly relevant for clinical outcomes (21).

The identification of efficient predictive biomarkers for ICIs-based therapies, such as PD-1/PD-L1 inhibitors, is useful for optimizing treatment, particularly in NSCLC, as evidenced by the results of the analyzed studies. A 2024 study assessed the predictive value of SNPs in the PD-L1 gene for patients with advanced NSCLC undergoing ICIs treatment. The study highlighted that the SNP rs822336 significantly predicted response to anti-PD-1/PD-L1 therapy in non-oncogene-addicted NSCLC. This SNP was found to induce PD-L1 expression through competitive allelic-specific binding of transcription factors C/EBPβ and NFIC. Silencing these transcription factors in NSCLC cell lines with different rs822336 genotypes showed differential regulation of PD-L1 expression. These findings suggest that rs822336, through its effect on PD-L1 expression, could serve as a biomarker for predicting the efficacy of PD-1/PD-L1-based immunotherapy in advanced NSCLC (22, 23).

In another study focused on advanced NSCLC patients receiving immunotherapy, SNP rs2297136 was found to have clinical significance. Analysis of clinical outcomes indicated that patients with the AA genotype of rs2297136 had a lower objective response rate (ORR) of 19.0%, compared to 29.0% in those with the AG/GG genotype. Additionally, the median PFS was 2.95 months for the AA genotype versus 5.30 months for the AG/GG genotype, and the median OS was 8.8 months for the AA genotype versus 18.4 months for the AG/GG genotype. These results suggest that the rs2297136 variant in the PD-L1 gene could be a potential biomarker for predicting clinical outcomes in patients receiving PD-1 blockade therapies (24). Further research on the polymorphisms rs822335 and rs2297136 revealed that patients with the TT genotype of rs822335 had a lower percentage of tumor cells expressing PD-L1 compared to those with the CC genotype. The study also noted a significantly higher risk of death in patients treated with chemotherapy compared to those treated with immunotherapy, suggesting that the rs822335 polymorphism may influence both PD-L1 expression and treatment response (25).

Additional investigations into the PD-L1 gene’s 3’-untranslated region (3’UTR) revealed that the rs4143815 GG and rs4742098 AA variants were associated with lower PD-L1 expression and poorer prognosis (26). In contrast, the rs4143815 GG variant was linked to higher PD-L1 expression, emphasizing the complex relationship between genetic variants and PD-L1 expression in cancer (27).

**Figure 2:**
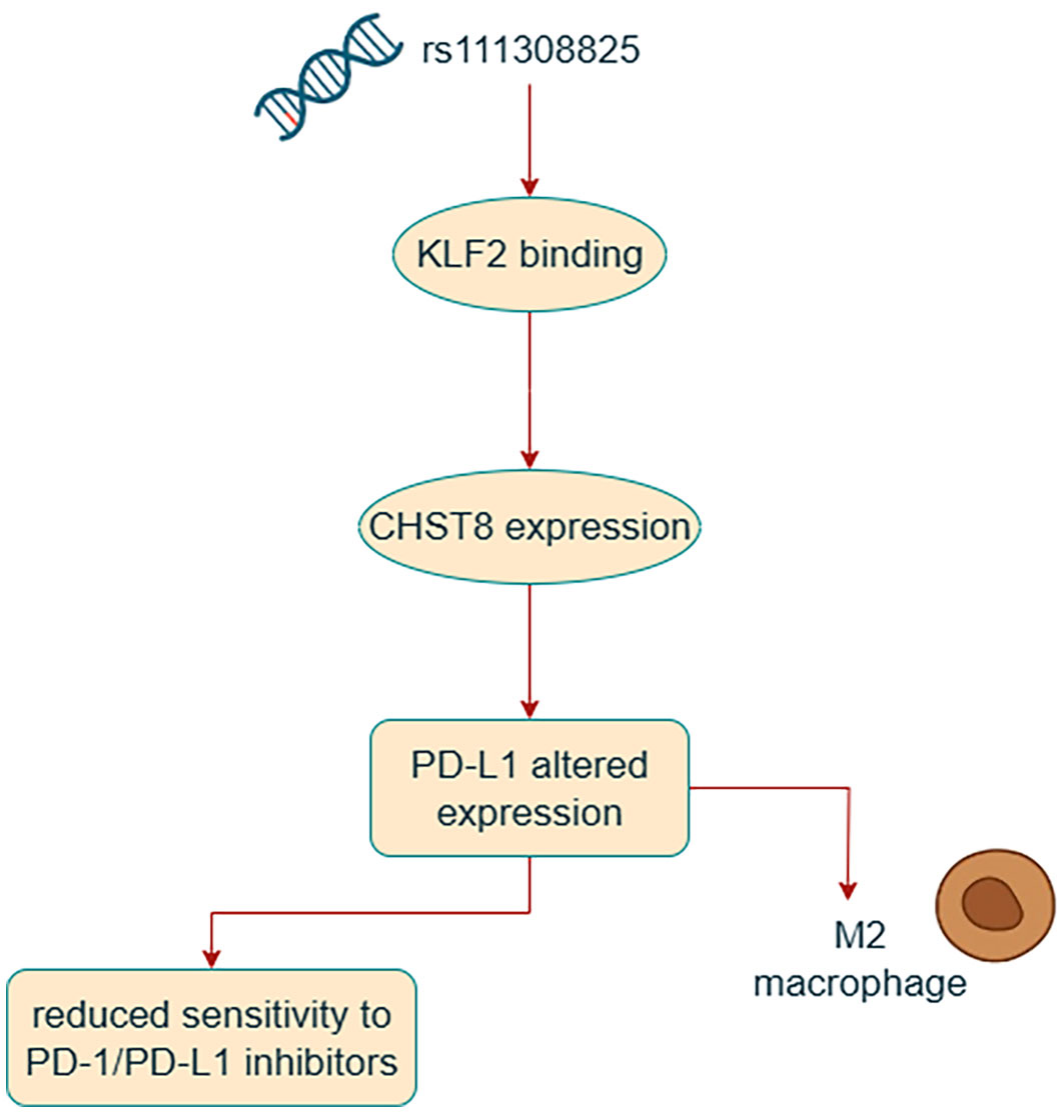
Graphical representation of the impact of rs111308825 on sensitivity to PD-1/PD-L1 Inhibitors.

One of the most significant loci identified in the PD-L1 gene was rs111308825, located in the enhancer region on chromosome 19q13.11. This SNP was found to impair KLF2 binding, leading to reduced expression of carbohydrate sulfotransferase 8 (CHST8). Tumor cells expressing CHST8 were shown to suppress T-cell activation and loss of CHST8 attenuated tumor growth in a syngeneic mouse model. Moreover, CHST8 is involved in the sulfation of PD-L1 and its homologs, contributing to the enrichment of M2-type macrophages in the tumor microenvironment. Tumors with low CHST8 expression demonstrated a better response to immunotherapy, supporting the clinical significance of rs111308825 in predicting immunotherapy efficacy (28) (**Figure 2**).

Functionally, PD-L1 variants often localize in promoter or 3′UTR regions: rs822336 modifies binding of transcription factors (C/EBPβ, NFIC), regulating PD-L1 transcription, while rs2297136 alters microRNA binding sites, affecting mRNA stability. Other SNPs, such as rs4143815 and rs4742098, further exemplify how allelic differences can modulate PD-L1 expression and the extent of tumor immune evasion.

### Other gene polymorphisms

ATM (Ataxia-telangiectasia mutated) is a gene involved in the DNA damage response, particularly in delaying the cell cycle after double-strand breaks (DSBs). It is known that ATM inhibition can increase DNA damage and activate the interferon response, thus modulating the TME and the efficacy of immunotherapy (29). In addition, some ATM SNPs are associated with increased gastrointestinal toxicity. Indeed, several studies have examined the correlation between ATM gene polymorphisms and therapy-induced adverse effects. A study indicated that patients homozygous for the ATM2 haplotype (rs4585*T, rs189037*A, rs227092*T, rs228590*C, and rs664677*T) are more likely to experience high-grade gastrointestinal toxicity compared to patients homozygous for the ATM1 haplotype. ATM gene SNPs predict regimen-related gastrointestinal toxicity in patients allografted after reduced conditioning (30).

Many of these variants have functional implications, since they may alter transcription factor binding sites in promoter regions or splicing efficiency, thereby influencing ATM protein levels and downstream DNA repair activity.

PTPN11 encodes a protein that is part of the protein tyrosine phosphatase (PTP) family, which regulates various cellular processes including cell growth, differentiation, mitotic cycle, and oncogenic transformation. A specific variant, 333-223A>G, has been associated with elevated transaminases and thyroid disorders (hypothyroidism or hyperthyroidism) in patients undergoing immunotherapy. Furthermore, a variant in the IFNG gene (1616T>C) has been linked to renal toxicity of any grade (31).

A genetic variant in the PTPN22 gene (R620W, rs2476601), which encodes a protein that belongs to the PTP family, contributes to the risk of autoimmunity by allowing increased T-cell receptor (TCR) signalling and activation in autoreactive T cells. This may potentially expand the pool of autoreactive T cells and predispose individuals to an inflammatory phenotype (32).

Regarding irAEs, several interesting associations have been identified. Three polymorphisms—rs16906115 near IL7, rs75824728 near IL22RA1, and rs113861051 on 4p15—have been linked to irAEs. The variant near IL7 is colocalized with the acquisition of a new cryptic exon for IL7, a regulator of lymphocyte homeostasis. Patients carrying the germline variant of IL7 showed increased lymphocyte stability after initiating ICIs, which correlated with improved survival (33).

Additionally, several other genes, including MTHFD2, SLC5A1, NT5DC4, AIRE, NKG2, MIF, M6A, MAGE-A3, FGFR-4, HLA-G, HLA-DQ1, CTSW, MHCII, CTSS, FCGR3B, ERAP 1-2, 4p15, and IL22RA1, are involved in regulating adverse reactions and therapeutic outcomes (34–42).

Many of these variants have defined mechanistic implications. For example, IL7 rs16906115 enhances lymphocyte stability following ICIs, explaining its dual role in irAEs and survival. HLA-DQA1 and HLA-G polymorphisms reshape antigen presentation and the tumor microenvironment, while CTSW rs3903072 is associated with greater tumor-infiltrating lymphocyte activity in breast cancer. Likewise, FGFR4 rs351855 accelerates oncogenic signaling and progression, and ERAP1/2 variants influence peptide processing and MHC presentation, thereby modulating the effectiveness of checkpoint blockade.

However, the available data in the literature still require further investigation.

The findings summarized in this review are presented in **Table 1** and **Table 2**.

**Table 1:** Correlation between single nucleotide polymorphism (SNP) with toxicities and survival outcomes in CTLA-4, PD-1 and PD-L1 genes.

Gene	SNP / Variant	Cancer type	Clinical association (Toxicity)	Reference
CTLA-4	rs733618, rs4553808, rs11571317, rs5742909, rs231775, rs3087243, rs7565213	Not specified	No significant association with severe autoimmune reactions (grade III-IV)	(14)
PD-L1	p8293 C>A (rs2890658), C>T (rs2297136), G>C (rs4143815)	Not specified	Reduced risk of irAEs; p8293 C/A VS C/C	(19)
PD-1	PD1.3 G>A, PD1.5 C>T, PD1.6 G>A, PD1.7 T>C, PD1.10 C>G, 804C>T	Melanoma / Renal / Not specified	Reduced likelihood of any grade treatment-related toxicity; PD1.6 G associated with severe irAE	(19, 20, 31)

Gene	SNP / Variant	Cancer type	Clinical association (Outcome)	Reference
CTLA-4	TT-genotype -1722T>C, GG-genotype CT60G>A, TT-genotype Jo27T>C, G-genotype Jo31G>T	Melanoma	Improved overall survival	(10)
CTLA-4	B7-2 rs2681416 Avs G	Colon	Higher risk of colon VS rectal cancer; promotes Th17 infiltration	(11)
CTLA-4	rs733618, rs4553808, rs11571317, rs5742909, rs231775, rs3087243, rs7565213	Not specified	TACCGGG haplotype: possible non-response; TGCCAGG haplotype: possible response	(14)
PD-1	PD1.3 G>A, PD1.5 C>T, PD1.6 G>A, PD1.7 T>C	Melanoma	T-allele dose-dependent positive trend in OS; PD1.3 associated with longer PFS	(17, 19)
PD-L1	rs4143815 GG, rs4742098 AA	Lung	Low PD-L1 expression -> poor prognosis; high expression -> better prognosis	(23, 27)
PD-L1	rs111308825	Breast	Low-CHST8 tumors -> better response to ICB	(28)

**Table 2:** Correlation between single nucleotide polymorphism (SNPs) and toxicities and survival outcomes in other relevant genes.

Biological function	Gene	SNPs	Cancer	Toxicity
Cytokines/Immune Modulators	IFNG	1616T>C	Not specified	Rheumatological toxicity (any grade) (31)
	IL-7	rs16906115	Not specified	Predictive of downstream irAEs and improved survival (33)
	IL22RA1	rs75824728	Not specified	All-grade irAEs (33)
	IL74p15	rs113861051	Not specified	All-grade irAEs (33)
	MIF	Rs755622	Glioblastoma	Increase in immune microenvironment signaling (37)
DNA Damage / Repair Pathways	ATM	rs4585 T/G, rs189037 A/G, rs227092 T/G, rs228590 C/T, rs664677 T/C	Not specified	Homozygous ATM2 haplotype increases high-grade gastrointestinal toxicity; ATM inhibition affects TIME and immunotherapy efficacy (29, 30)
Protein Tyrosine Phosphatases / Signal Regulators	PTPN11	333-223A>G	Not specified	Elevated transaminases; hypo/hyperthyroidism (any grade) (31)
	PTPN22	Rs2476601	Not specified	Autoimmunity risk due to increased TCR signating (32)

Biological function	Gene	SNPs	Cancer	Outcomes
Cytokines / Immune Modulators	AIRE	rs1055311, rs1800520, Rs1800522	Not specified	Increased T-cell clonotypes specific for MAGE-1 (34)
	NKG2D / NKG2A	rs1049172, rs1983526	AML	Better immunotherapy response (36)
	MIF	Rs755622	Glioblastoma	Immune microenvironment signating (37)
Metabolic / Epigenetic Regulators	MTHFD2, SLC5A1, NT5DC4 N6-	Multiple	Prostate, lung adenocarcinoma, ESCA	Reprogramming, immune evasion, poor survival (35)
	methylade nosine (m6A)	TCGA data	Esophageal/ Stomach	Low m6A scores trigger immune response via neoantigen load (38)
Tumor Microenvironment / Antigen Presentation	MAGE-A3	rs5970360, rs5925210, rs5970361, rs5925211, rs35123853	Not specified	EGFR mRNA expression correlated with SNP genotypes (39)
	HLA- DQA1	rs1129735, rs3135344	Melanoma / Prostate	Modifies tumor microenvironment (42)
	HLA-G	rs13193697, rs9260555	Melanoma / Prostate	Modifies tumor microenvironment (42)
	ERAP1-2	rs6875109, rs2927611, rs62376450	Melanoma / Prostate	Modifies tumor microenvironment (42)
	CTSS	rs1053732 rs141935877	Melanoma/Prostate	Modifies tumor microenvironment (42)
	FCGR3B	Rs6671847	Prostate	Modifies tumor microenvironment (42)
Signating/Other	FGFR4	Rs351855	Not specified	Poor prognosis, accelerated progression (40)
	CTSW	Rs3903072	Breast	Regulates tumor-infiltrating lymphocytes; positively correlated with survival (41)
	PTPN22	Rs2476601	Not specified	Autoimmunity risk (32)

## Discussion

Numerous studies have investigated the relationship between SNPs, disease prognosis, and treatment-related adverse effects, influencing in some cases clinical practice. For example, the relationship between the degradation rate of 5-fluorouracil (5-FU) and genetic polymorphisms in the DPYD, TSER, MTHFR A1298T, UGT1A1 and C677T genes has been studied. The results led to the development of predictive models, in particular, for the prevention of 5-FU and CPT-11 toxicity, results subsequently incorporated into clinical practice for the treatment of patients diagnosed with gastrointestinal neoplasia (43, 44). In patient candidate to 5-FU and/or CPT-11 treatment is nowadays considered mandatory the analysis, through simple blood sample, of5-FU metabolism, and a genomic panel, for the evaluation of the enzymatic activity of DYPD, UGT, and other genes. This pharmacogenomic analysis, which precedes the chemotherapy start, is useful to prevent serious adverse reactions.

However, although the role of SNPs is demonstrated before chemotherapy and other drugs, few data are available in the context of immunotherapy.

Immunotherapy, widely adopted for treating various cancer types either as monotherapy or in combination with chemotherapy or targeted therapies, has introduced a diverse array of potential adverse events, which can be difficult to predict and manage. While being able to predict the response to ICIs and understand their long-term outcome remains a priority goal.

Clinical trials have shown a wide range of 5-year OS rates for patients undergoing ICI treatment, depending on cancer type, treatment line, and patient characteristics. Additionally, a significant proportion of patients experience disease progression within months of starting ICI therapy (45). These challenges underscore the urgent need for reliable predictive biomarkers to guide treatment decisions and improve patient outcomes.

In this context, SNPs in immune checkpoint genes such as CTLA-4, PD-1, and PD-L1 have emerged as potential biomarkers for predicting both the efficacy and toxicity of immunotherapy. Several studies have identified polymorphisms in the CTLA-4 gene that may influence treatment outcomes. For example, polymorphisms like -1722T>C and CT60G>A have been associated with reduced rates of severe adverse events and improved overall survival in patients treated with Ipilimumab. Furthermore, variants such as Jo27T>C and Jo31G>T are linked to enhanced survival, suggesting that CTLA-4 polymorphisms could serve as valuable predictive biomarkers for immunotherapy. Additional variants like rs2681416 in B7–2 and CTLA-4c.-1661A>G have been implicated in both cancer susceptibility and immune cell infiltration, suggesting that these polymorphisms could influence cancer risk and immune responses within the tumor microenvironment.

Notably, some of these variants act through transcriptional mechanisms. For instance, CTLA-4c.-1661A>G creates a novel binding site for the transcription factor C/EBPβ, leading to increased CTLA-4 expression and enhanced inhibitory signalling in T cells. This mechanistic insight helps explain why carriers of this variant may display altered immune responses and cancer susceptibility (12, 13).

While these findings are promising, further validation is required to determine their clinical utility in practice.

Similarly, SNPs in the PD-1 gene have shown potential as predictive biomarkers for response to anti-PD-1 therapies. For example, the PD1.3 (rs11568821) polymorphism has been associated with better clinical outcomes in patients with metastatic melanoma undergoing anti-PD-1 therapy, indicating its utility in predicting therapeutic efficacy. On the other hand, variants like PD1.6 (rs10204225) are associated with an increased incidence of immune-related adverse events (irAEs), highlighting the importance of these genetic markers in monitoring treatment safety.

Mechanistically, certain PD-1 variants may affect receptor expression or signaling. For instance, rs11568821 disrupts a RUNX1 binding site in intron 4, which may impair proper transcriptional regulation of PD-1, whereas rs2227981 in the promoter region has been linked to altered PD-1 expression levels. These regulatory effects could contribute to differences in T-cell exhaustion and immune checkpoint sensitivity among patients (17, 46).

Identifying such SNPs could aid in personalizing treatment by predicting which patients are most likely to benefit from immunotherapy and which are at higher risk of adverse effects. Similarly, polymorphisms in the PD-L1 gene, such as rs822336 and rs2297136, have been found to affect responses to PD-1/PD-L1 blockade therapies, particularly in NSCLC. These polymorphisms may serve as predictive biomarkers for immunotherapy efficacy. Variants like rs4143815 GG and rs4742098 AA, which are associated with differential PD-L1 expression, highlight the complex relationship between genetic variations and PD-L1 expression in tumors.

Importantly, some of these polymorphisms exert their effects through transcriptional and post-transcriptional mechanisms. For example, rs822336 has been shown to alter transcription factor binding (C/EBPβ, NFIC), thereby modulating PD-L1 expression. Likewise, SNPs in the 3′ untranslated region (e.g., rs4143815, rs4742098) may affect mRNA stability and microRNA interactions, resulting in differential PD-L1 expression. These mechanisms provide a functional explanation for the observed variability in immunotherapy outcomes and underscore the importance of integrating molecular biology with clinical genetics (22, 47).

These variations could provide insights into prognosis and treatment response, underscoring the potential utility of PD-L1 genetic markers in clinical practice. Together, the various studies analyzed in the review provide evidence that PD-1 gene polymorphisms may serve as predictive biomarkers for both the efficacy of anti-PD-1 therapies and the risk of irAEs across various cancer types. These findings underline the potential for using PD-1 SNPs to guide clinical decision-making and personalize immunotherapy.

Moreover, genetic variants in genes such as ATM, PTPN11, and PTPN22, which regulate immune responses and T-cell activation, have been linked to treatment-related adverse effects. Therefore, the study of ATM SNPs could give us interesting data on the response to immunotherapy and possible immune-related gastrointestinal toxicities, while variants in PTPN11 and PTPN22 have been connected to thyroid dysfunctions and enhanced autoimmune responses.

Variants in these pathways often act by modulating DNA repair efficiency, T-cell receptor signaling, or cytokine receptor expression, thereby indirectly shaping immune activation and tolerance.

These findings are critical for identifying patients at risk for autoimmune reactions during immunotherapy, enabling more tailored management approaches.

Certainly, our review has some limitations. First of all, it a systematic review and no metanalysis was performed to compare the studies identified. Secondly, we did not search for SNPs detected on databases such as dbSNP or PharmGKB.

We strongly believe that the complexity of immune responses and immune evasion mechanisms in cancer necessitates further large-scale studies to validate these SNPs and their clinical applications.

Anyway, the aim of this systematic review was to bring out the most recent data to create a panel of SNP variants that can help clinicians in their therapeutic choices. To integrate this review into the broader framework of precision medicine to highlight the importance of personalizing treatment strategies based on molecular profiles. As cancer treatment should involve comprehensive multi-omic profiling, including genomics, transcriptomics, proteomics and immunomics (48).

In this context, our research group is starting a prospective multicentric trial focusing on the most important SNPs before starting ICIs in all cancer subtypes aiming to predict response and adverse events.

We strongly believe that, in the precision medicine era, a comprehensive approach combining genetic, clinical, and immunological data will be crucial for optimizing immunotherapy, minimizing adverse effects, and improving patient outcomes.

## Conclusions

SNPs in key immune checkpoint genes such as CTLA-4, PD-1, and PD-L1 have emerged as promising biomarkers for predicting both cancer susceptibility and the efficacy of immunotherapy. Variants in these genes can influence immune responses, treatment outcomes, and the risk of developing irAEs, highlighting the potential for personalized cancer therapy.

The study of SNPs, therefore, may serve as a starting point that could lead to a change in clinical practice, in the approach to patients undergoing immunotherapy treatment. We expect that a detailed study of the various SNPs will be useful in the context of both localized and extensive disease and that it may be extendable to various types of immunotherapeutic drugs.

Further research and large-scale validation are needed to establish their clinical utility and guide decision-making in immunotherapy. As the field of genetic biomarkers in immunotherapy continues to evolve, integrating these findings into clinical practice could enhance the precision and effectiveness of cancer treatment strategies.
